# Supplementary material for: A Global Screen for Assembly State Changes of the Mitotic Proteome by SEC-SWATH-MS
Source: Cell Syst. 2020 Feb 26;10(2):133–155.e6. doi: 10.1016/j.cels.2020.01.001 (PMC7042714; doi:10.1016/j.cels.2020.01.001)

O95819 | M4K4_HUMAN | MAP4K4 HGK KIAA0687 NIK
Monomer MW [kDa]: 142.101 Monomer expected elution fraction: 37

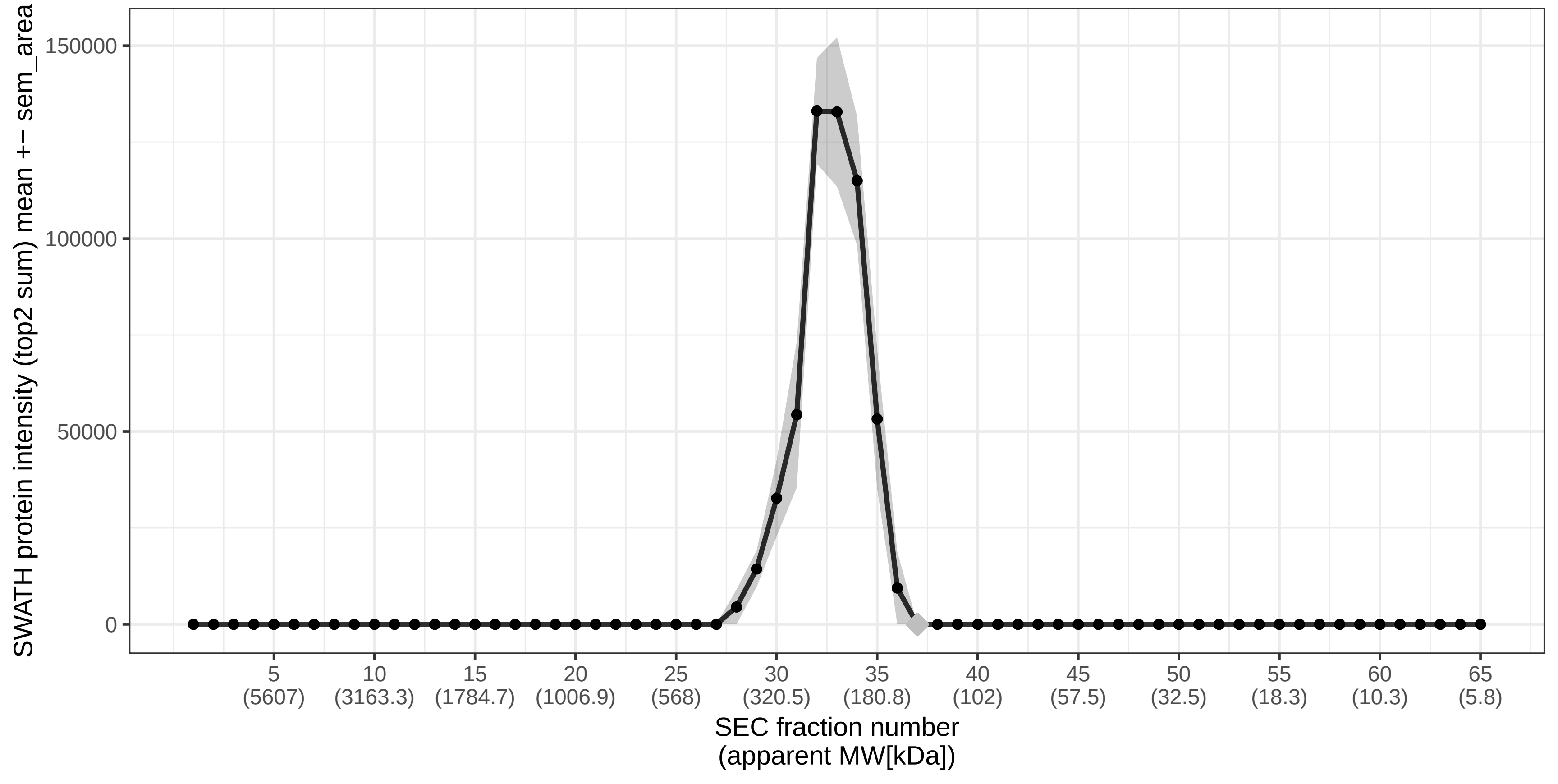

Supplement: Data S1. SEC-SWATH-MS Protein Chromatograms, Related to Figure 1 [file mmc6.zip › SECchrom_O95819_M4K4_HUMAN_MAP4K4_HGK_KIAA0687_NIK.pdf]
